# Data on autophagy markers and anti-candida cytokines expression in mice in response to vaginal infection of *Candida albicans*

**DOI:** 10.1016/j.dib.2018.03.006

**Published:** 2018-03-09

**Authors:** Ankit Shroff, Roicy Sequeira, Vainav Patel, K.V.R. Reddy

**Affiliations:** a Division of Molecular Immunology & Microbiology (MIM), National Institute for Research in Reproductive Health (NIRRH), J. M. Street, Parel, Mumbai 400012, India; b Department of Biochemistry & Virology, National Institute for Research in Reproductive Health (NIRRH), J. M. Street, Parel, Mumbai 400012, India

**Keywords:** Autophagy markers, ATG5, Vaginal infection, *Candida albicans*, Cytokines

## Abstract

The data presented here are related to the research article entitled “Knockout of autophagy gene, ATG5 in mice vaginal cells abrogates cytokine response and pathogen clearance during vaginal infection of *Candida albicans*” (Shroff et al., 2018) [1]. The cited research article describes the role of autophagy in host immune response against *C. albicans* infection of mice vagina. In this data report wild-type C57BL/6 mice were infected intravaginally with *C. albicans*. Vaginal cells were analyzed for the expression of autophagy marker genes LC3 & ATG5 and lysosome marker LAMP1 at the transcript and protein level. Vaginal lavages were also obtained from these infected mice. The levels of pro-inflammatory and T-helper cell related cytokines were determined in these lavages.

## Specifications Table

**Table:** 

Subject area	Biology
More specific subject area	Immunology, Microbiology and Molecular Biology
Type of data	Figures and graphs
How data was acquired	1. Reverse Transcriptase PCR (RT-PCR) followed by agarose gel electrophoresis, imaged on Gel Documentation Unit (Bio-Rad, USA) 2. Quantitative PCR (qPCR) on CFX 96 real-time PCR system (Bio-Rad, USA) using SYBR Green chemistry 3. Western blot using Bio-Rad equipment 4. Multi cytokine analysis with data acquisition on BD Acuri C6 flow cytometer.
Data format	Analyzed
Experimental factors	Vaginal infection of mice with *Candida albicans* and collection of lavages
Experimental features	1. RT-PCR and qPCR to analyze the transcript levels of LC3, ATG5 and LAMP1. 2. Western blotting to analyze the protein levels of LC3, Atg5 and LAMP1. 3. Multi cytokine analysis to detect the levels of G-CSF, IL-1α, IL-1β, IL-6, IL-10, IL-17A, IL-22, IL-23p19 and TNF-α
Data source location	Division of Molecular Immunology and Microbiology, National Institute for Research in Reproductive health (NIRRH), Mumbai, India.
Data accessibility	Data is within this article

## Value of the data

• This is the first study on the up-regulation of autophagy markers and cytokines related to anti-Candida activity during in-vivo vaginal infection of wild-type C57BL/6 mice.
• The data describes the expression of autophagy marker genes in vaginal cells of wild-type C57BL/6 mice after intravaginal infection of *C. albicans*.
• This data detects the levels of anti-candida related cytokines in the vaginal lavages of wild-type C57BL/6 mice throughout the duration of infection.

## 1 Data

### 1.1 Vaginal *C. albicans* infection induces the expression of autophagy marker genes

**Fig. 1:**
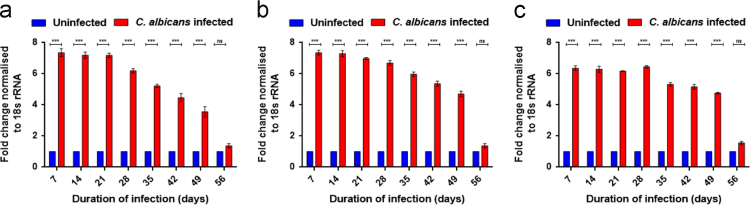
**Transcript level analysis of autophagy marker genes in wild-type C57BL/6 mice infected with*****C. albicans***. qPCR of LC3 (a), ATG5 (b) and LAMP1 (c) transcripts in vaginal cells isolated from wild-type C57BL/6 mice infected vaginally with *C. albicans* for different time points and their respective uninfected mice. 18S rRNA was used as the reference gene. Lane 1: 50 bp ladder, Lane 2: 7 days post-infection, Lane 3: 14 days post-infection, Lane 4: 21 days post-infection, Lane 5: 28 days post-infection, Lane 6: 35 days post-infection, Lane 7: 42 days post-infection, Lane 8: 49 days post-infection, Lane 9: 56 days post-infection, Lane 10: No template control (*p* values: ***p < 0.001, ns: not significant).

We determined the levels of LC3, ATG5 and LAMP1 in *C. albicans* infected and uninfected mice of different strains at transcript and protein level. We observed that wild-type C57BL/6 mice infected with *C. albicans* showed a distinct increase in the levels of LC3 transcript (Fig. S1a). This increase was about 7-fold 7 days post*-C. albicans* infection and was maintained at significantly (*p* < 0.001) high levels till 49 days post-infection (Fig. 1a). Apart from LC3, there was an upregulation in the expression of ATG5 transcripts (Fig. S1b). This upregulation resulted in a 7-fold increase in ATG5 transcripts in the infected mice at 7 days post-infection. The transcript levels returned to the levels of uninfected mice 56 days post-infection (Fig. 1b). We also observed an escalation in the levels of LAMP1 transcripts in infected mice (Fig. S1c). This increment was 6-fold at 7 days post-infection and was observed at significant levels till 49 days post-infection (Fig. 1c).

**Fig. 2:**
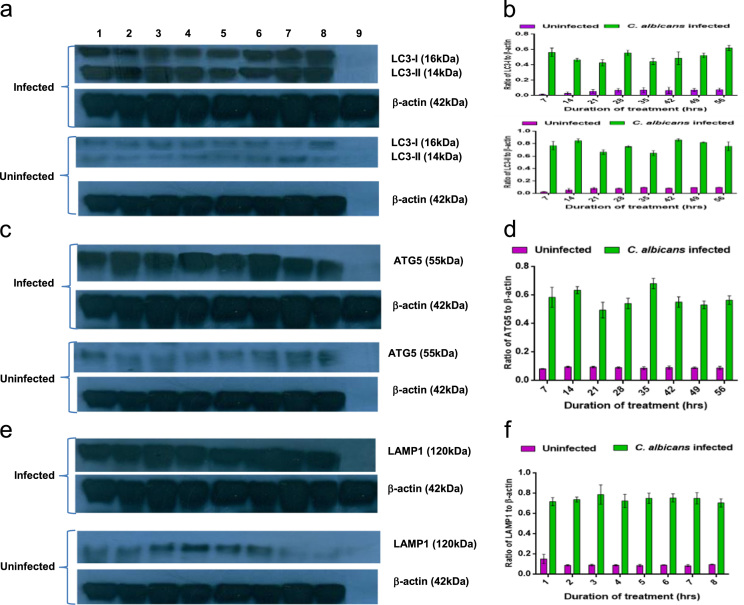
**Protein level analysis of autophagy marker genes in wild-type C57BL/6 mice infected with C. albicans.** Western blot and densitometry analysis of LC3 (14 kDa and 16 kDa) (a, b), ATG5 (55 kDa) (c, d) and LAMP1 (120 kDa) (e, f) in vaginal cells isolated from C57BL/6 mice infected vaginally with *C. albicans* for different time points and their respective uninfected mice. β-actin (42 kDa) was used as the reference gene. Lane 1: 7 days post-infection, Lane 2: 14 days post-infection, Lane 3: 21 days post-infection, Lane 4: 28 days post-infection, Lane 5: 35 days post-infection, Lane 6: 42 days post-infection, Lane 7: 49 days post-infection, Lane 8: 56 days post-infection, Lane 9: Primary antibody control (*p* values: ***p < 0.001, ns: not significant).

To confirm these results at the protein level, we carried out western blot analysis to detect the levels of LC3, ATG5 and LAMP1 in infected as well as uninfected C57BL/6 mice. LC3 levels increased significantly in *C. albicans* infected C57BL/6 mice as compared to the uninfected and infected mice (Fig. 2a). Densitometry results revealed that 7 days post-infection there was a 3 and 5 fold increases in LC3-I and LC3-II respectively. At 56 days post-infection, the levels of LC3-I and LC3-II returned to the levels of uninfected mice (Fig. 2b). Detection of protein levels of ATG5 in the vaginal cells of *C. albicans* infected C57BL/6 mice indicated an escalation in the expression of Atg5 in the infected mice as compared to the uninfected mice (Fig. 2c). This escalation on quantification revealed a 4-fold increase in the levels of Atg5 in the *C. albicans* infected mice as compared to the uninfected mice at 7 days post-infection. The levels declined slowly over the period of infection but remained at significantly higher levels (*p* < 0.001) till the infection is cleared (49 days) (Fig. 2d). LAMP1 protein expression also showed an increment in the infected mice as compared to the uninfected mice (Fig. 2e). Densitometry results showed 3-fold increment in LAMP1 levels at 7 days post-infection in infected mice as compared to the uninfected mice. This increment was sustained throughout the duration of infection (Fig. 2f).

### 1.2 Production of various anti-Candida cytokines is upregulated during vaginal infection of *C. albicans*

**Fig. 3:**
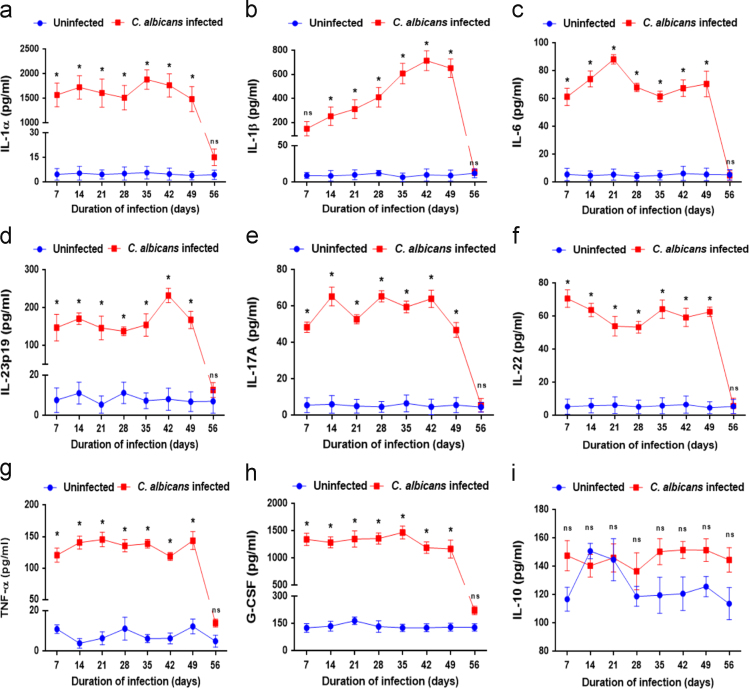
Levels of different cytokines in the vaginal lavages of wild type C57BL/6 mice at different time points during vaginal *Candida albicans* infection. The cytokines assayed include IL-1α (a), IL-1β (b), IL-6 (c), IL-23p19 (d), IL-17A (e), IL-22 (f), TNF-α (g), G-CSF (h) and IL-10 (i). Uninfected: mice with intravaginal PBS administration; *C. albicans* infected: mice with vaginal infection of *C. albicans* (p values: *p < 0.05, ns: not significant).

We checked the levels of cytokines in the vaginal lavages of wild-type C57BL/6 mice, which were intra-vaginally infected with *C. albicans* blastospores. IL-1αlevels in the vaginal lavages of *C. albicans* infected mice increased by 150 folds as compared to the uninfected mice at 7 days post-infection. The levels were measured to be 1500 ± 250 pg/ml throughout the duration of infection, which lasted for about 49 days (Fig. 3a). The levels of IL-1β in the vaginal lavages of infected mice revealed a 10-fold increase at 7 days post-infection and continued to rise and peaked to 600 ± 95 pg/ml at 6 weeks post-infection (Fig. 3b). IL-6 increased by 6-fold at 7 days post-infection and remained around 70 ± 10 pg/ml throughout the infection (Fig. 3c).

IL-23p19 increased by 15 folds at 7 days post-infection and reached 200 pg/ml 6 weeks post-infection (Fig. 3d). IL-17A levels were increased by 5 folds as compared to the uninfected mine and levels were measured to be 60 ± 10 pg/ml throughout the duration of infection (Fig. 3e). A 7 fold increase was observed in IL-22 levels at 7 days post-infection and the levels were measured to be about 60 ± 10 pg/ml throughout the infection (Fig. 3f). The levels of TNF-α were measured to be 125 ± 25 pg/ml in infected mice as compared to uninfected mice (10 pg/ml) and the levels reduced by the end of day 56 (Fig. 3g). G-CSF levels were increased by 100-folds at 7 days post-infection and remained at similar levels till 49th day post-infection (Fig. 3h). IL-10 is the only cytokine that did not increase in response to *C. albicans* infection and the levels (130 ± 25 pg/ml) were comparable between infected and uninfected mice (Fig. 3i. These cytokine values of C. albicans infected wild-type C57BL/6 mice were used for comparison with the cytokine values in conditional autophagy knockout mice [1].

### 1.3 Polymorphonuclear leucocytes (PMNLs) infiltration escalated in response to *C. albicans* infection

**Fig. 4:**
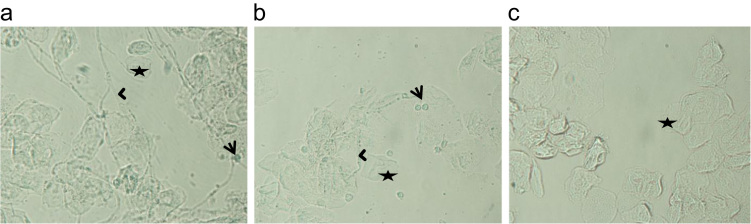
**Photomicrographs of vaginal lavages showing*****Candida albicans*****hyphae and polymorphonuclear leucocytes.** The vaginal lavages of *C. albicans* infected wild-type C57BL/6 mice (a–c) and PR-ATG5-KO mice (d–f) *were* evaluated on day 7 (a, d), day 28 (b, e) and day 56 post-infection (c, f). Arrow with tail: polymorphonuclear leucocytes, Star: Cornified vaginal epithelial cells, Arrow without tail: *C. albicans* hyphae (Magnification: 40×).

Wild-type C57BL/6 mice vaginal lavage smears displayed the presence of PMNLs in the vaginal lavage 7 days post-infection (Fig. 4a). A dense network of *C. albicans* hyphae was observed around cornified epithelial cells 1 week post-infection. PMNL number significantly surged post 4 weeks of infection (Fig. 4b). PMNLs and *C. albicans* hyphae were not observed in the vaginal lavage smears after 8 weeks of infection (Fig. 4c).

## 2 Experimental design, materials and methods

### 2.1 Ethics statement

Institutional Animal Ethics Committee of National Institute for Research in Reproductive Health (NIRRH) (IAEC project no: 05/08/RB/25/12) approved the study. All animal procedures were performed as per the guidelines of Committee for the Purpose of Control and Supervision of Experiments on Animals (CPCSEA). All infection experiments were carried out at the animal house facility of Haffkine Institute, Parel, and Mumbai after obtaining the necessary permissions from NIRRH Animal Ethics Committee and Haffkine Institute Animal Ethics Committee.

### 2.2 Procurement of animals

Two pairs of wild type C57/BL6 mice (60 days old, 20 ± 2 g body Wt.) were procured from Jackson Laboratories, USA. All these mice were maintained at 24 ± 2 °C under 12/12 h dark/light cycles at the animal house facility of NIRRH. The mice had access to standard pellet diet and water ad libitum throughout the study.

### 2.3 *Candida albicans* procurement and culture

*Candida albicans* (strain: 3153A) was obtained as a kind gift from Dr. Paul Fidel Jr., Department of Microbiology, Immunology and Parasitology, New Orleans School of Medicine, Los Angeles, USA. The strain was grown on HiChrome Candida agar (Himedia, India). Individual colonies were inoculated in Sabouraud's broth (Himedia, India) and incubated at 30 °C for 16 h in a shaker incubator. Glycerol stocks of candida cultures were stored at − 80 °C until experimentation. For vaginal infection, *C. albicans* was seeded in Sabouraud's broth and incubated for 16 h at 30 °C in a shaker incubator. Blastospores of *C. albicans* were harvested and re-suspended in phosphate buffered saline (PBS). Viable *C. albicans* blastospores were stained with Trypan-blue dye exclusion staining and counted using phase contrast microscope (Olympus, Japan) (Magnification × 40).

### 2.4 Vaginal infection of mice with *Candida albicans* and collection of lavages

To induce pseudo-estrous stage in mice, 100 µg of 17-β estradiol was subcutaneously injected in female mice (6–8 week old) 72 h prior to *C. albicans* infection. These injections were repeated once every week to maintain pseudo-estrous stage during the course of this study. These mice were used for vaginal Infection of *C. albicans* as per the protocol described previously [2]. Briefly, single dose of *Candida albicans* blastospores (5 × 10^6^ cells/mice) were intra-vaginally administered to pseudo-estrous mice. Control mice received only the vehicle, PBS (Phosphate buffered saline). The infections were carried out in wild-type C57BL/6 mice consisting of five mice in each group. Vaginal lavages were collected from infected and control mice at weekly intervals using 50 µl of PBS administered intra-vaginally.

### 2.5 Total RNA extraction from vaginal cells

Mice vaginal tissue was excised, cleaned from blood stain and damp, washed in PBS, and cut in to small pieces (4–5 mm) placed in a sterile multi-well plate containing 0.5 ml/well of Dispase (1.7 U/ml) on a shaking platform overnight at 4 °C. The cells thus obtained were suspended in 10× trypsin-EDTA and incubated at 37 °C for 10 min. Cells were centrifuged, washed with PBS and re-suspended in complete DMEM with 10% FBS. Cells were sheared using syringe with 21-gauge needle, washed with PBS and enumerated by trypan blue dye exclusion. Equal numbers of cells from each treatment group were treated with TRIzol reagent (Invitrogen, USA) to extract total cellular RNA. The extracted RNA was isolated using phenol-chloroform method and treated with DNase I (Sigma-Aldrich, USA). The concentration of the RNA was determined using Synergy H1 Microplate Reader (Bio-tek, USA). Purified RNA (1 µg) was subjected to first strand cDNA synthesis using the iScript cDNA synthesis kit according to the manufacturer's instructions (Bio-Rad, USA).

### 2.6 Reverse transcriptase PCR (RT-PCR)

The cDNA (1 μl) was amplified using 0.1 μM of each primer, 1 U of Taq DNA polymerase (Invitrogen, USA), PCR buffer with 1.5 mM MgCl_2_ (Roche Diagnostics, Germany), and 0.25 mM dNTPs (Genei, India) in a 20 μl reaction volume in Veriti Thermal Cycler (Applied Biosystems, USA). The gene specific primers were designed using Primer blast tool, National Center for Biotechnology Information (NCBI) website. The primer pairs with the lowest self-complementarity and GC contents were selected for the study and procured from Sigma-Aldrich, USA.The primers used are: LC3 (F: 5′-CCCACCAAGATCCCAGTGAT-3′; R: 5′-CCAGGAACTTGGTCTTGTCCA-3′), ATG5 (F: 5′-GTGTGAAGGAAGCTGACGCTTT-3′; R: 5′-GGAGGGTATTCCATGAGTTTC-3′) and LAMP-1(F:5′-ATGGCCAGCTTCTCTGCCTCC-3′;R:5′-ACAGTGGGGTTTGTGGGCAC 3′) while the house housekeeping gene primers used were: 18s rRNA (F: 5′-GTAACCCGTTGAACCCCATT-3′;R:5′-CCATCCAATCGGTAGTAGCG-3′). Amplification conditions were as follows: initial denaturation at 95 °C for 15 s, followed by 39 cycles comprising denaturation at 95 °C for 30 s, annealing for 15 s at 58 °C for LC3, ATG5 and 18s rRNA and 64 °C for LAMP1, and extension at 72 °C for 45 s. The final extension was carried out for 5 min at 72 °C. The products were analysed on 2% (w/v) agarose gels (Thermo-Scientific, USA) prepared in Tris Borate EDTA (TBE) buffer. The gels were stained with ethidium bromide (0.5 mg/ml) and visualized under ultraviolet trans-illuminator. The product size was approximated using a 50-bp DNA ladder (Genetix, USA). Negative controls did not contain cDNA in the reaction mixture. The gel pictures were recorded using gel documentation unit (Bio-Rad, USA).

### 2.7 Quantitative PCR

The target genes were amplified using conditions same as described for RT-PCR. LC3, ATG5 LAMP-1 and 18s rRNA transcript levels were determined by CFX 96 real-time PCR system (Bio-Rad, USA) using SYBR Green chemistry (Bio-Rad, USA). The fluorescence emitted at each cycle was collected after the extension step of each cycle. The homogeneity of the amplicons was verified by studying the melt curve. Mean Ct values generated in each experiment were used to obtain fold change by the ΔΔCt method using the CFX Manager software (Bio-Rad, USA). 18s rRNA was used as reference gene to normalize the levels of LC3, ATG5 and LAMP-1 genes in the above method [3].

### 2.8 Total protein extraction from vaginal cells and western blotting analysis

Mice vaginal tissue was excised, cleaned from blood stain and damp, washed in PBS, and cut in to small pieces (4–5 µM) placed in a sterile multi-well plate containing 0.5 ml/well of Dispase (1.7 U/ml) on a shaking platform overnight at 4 °C. The cells thus obtained were suspended in 10× trypsin-EDTA and incubated at 37 °C for 10 min. Cells were centrifuged, washed with PBS and re-suspended in complete DMEM with 10% FBS. Cells were sheared using syringe with 21-gauge needle, washed with PBS and enumerated by trypan blue dye exclusion. Proteins were extracted from equal number of cells from each treatment group using RIPA with protease inhibitor cocktail (Sigma, USA). The homogenates were centrifuged at 12,000 *g* for 30 min at 4 °C and the supernatants were collected. The concentration of total protein was determined using BCA protein estimation kit as per manufacturer's instructions (Pierce, USA). 10 μg of the samples were heated at 95 °C for 15 min in Laemmle buffer (Bio-Rad, USA). Electrophoresis was carried out on 12% (v/v) sodium dodecyl sulphate polyacrylamide gel. Each lane was loaded with 10 μg of protein. The separated proteins were transferred to a polyvinylidene fluoride (PVDF) membrane (Merck-Millipore, USA), followed by blocking with 5% (w/v) non-fat dry (NFD) milk powder (Bio-Rad, USA) in PBS (0.01 M, pH 7.2) at 25 °C for 1hr. The blots were incubated at 4 °C overnight with rabbit anti-human LC3 antibody {(Cell Signalling Technologies Cat. No. 3868, USA) (1:1,000 dilution in 5% (w/v) Bovine serum albumin (Sigma Aldrich, USA) in PBST (0.1% v/v Tween-20}, rabbit anti-human Atg5 antibody {(Abcam Cat no. ab108327) (1:1,000 dilution in 5% (w/v) Bovine serum albumin (Sigma Aldrich, USA) in PBST (0.1% v/v Tween-20)} and rabbit anti-β-actin antibody {(Abcam, Cat. No. ab8227, USA) (1:1000 dilution in 5% (w/v) Bovine serum albumin (Sigma Aldrich, USA) in PBST (0.1% v/v Tween-20)}. The blots were washed four times with PBST for 15 min each and then incubated for 1hr at 25 °C with horseradish peroxidase (HRP)-conjugated goat anti-rabbit secondary antibody {(Cell Signalling Technologies; Cat. No. 7074, USA) (dilution 1:1,000 in 5% NFD milk powder (Bio-Rad, USA) in PBST)}. The blots were then washed 8 times with PBST for 15 min each and signals were detected using the ECL Advance chemiluminescence detection system (GE Healthcare, USA).

### 2.9 Cytokine analysis

The vaginal lavages collected from infected and uninfected mice strains were subjected to multi cytokine analysis (G-CSF, IL-1α, IL-1β, IL-6, IL-10, IL-17A, IL-22, IL-23p19) and TNF-α using Aimplex Premixed Analyte kit as per the manufacturer's protocol (YSL Bioprocess Development Co). The assay is based on the principle of sandwich ELISA. Briefly, each bead in a population is conjugated with capture antibodies specific for one cytokine. This antibody traps the protein of interest from the sample. The amount of the analyte captured is detected via a biotinylated antibody against a secondary epitope of the protein, followed by streptavidin-R-phycoerythrin (PE) treatment. Concentrations of cytokines in the samples were determined by comparing the fluorescent signals of samples against a standard curve generated from serial dilutions of known concentrations of the analyte. The assay protocol consists of a 60-min incubation step to allow antigen capture by antibody conjugated bead, a 30 min incubation step for biotinylated-antibody detection of analyte and a 20 min streptavidin-PE incubation step. The fluorescence intensities of the beads were measured using BD Acuri C6 flow cytometer at the Department of Biochemistry and Virology, NIRRH. Each sample was tested in duplicate and the mean of the two samples was used for analysis. The data was processed using FlowJo software and each cytokine concentration was expressed in pg/mL.

### 2.10 Estimation of infiltrated Polymorphonuclear leucocytes (PMNLs)

Vaginal lavage (10 μl) was smeared on a clean glass slide and observed under phase contrast microscope (Olympus, Japan) (Magnification × 40). Images were captured with a digital camera. Ten random fields were viewed per slide and the numbers of leucocytes in filtered into the vaginal lumen were determined.

### 2.11 Statistical analysis

The data shown are representative results. Experimental values are represented as means ± SEM. For time based comparisons, *p* values were calculated using 2-way ANOVA followed by Sidak's multiple comparison tests. For comparisons between different strains of mice, p values were calculated by 2-way ANOVA followed by Turkey's multiple comparison tests using GraphPad PRISM 6 software, USA. Values for conditions having significant difference are shown as asterisks in figures. Results were considered significant if *p* value was < 0.05.

## Appendix A Supplementary material

Supplementary material **Figure S1: Transcript level analysis of autophagy marker genes in wild-type C57BL/6 mice infected with*****C. albicans***. Reverse Transcriptase PCR (RT-PCR) of LC3 (79 bp) (a), ATG5 (148 bp) (b) and LAMP1 (521 bp) (c) transcripts in vaginal cells isolated from wild-type C57BL/6 mice infected vaginally with *C. albicans* for different time points and their respective uninfected mice. 18s rRNA (150 bp) was used as the reference gene. Lane 1: 50 bp ladder, Lane 2: 7 days post-infection, Lane 3: 14 days post infection, Lane 4: 21 days post infection, Lane 5: 28 days post-infection, Lane 6: 35 days post-infection, Lane 7: 42 days post-infection, Lane 8: 49 days post-infection, Lane 9: 56 days post-infection, Lane 10: No template control.
