# Supplementary material for: Data on autophagy markers and anti-candida cytokines expression in mice in response to vaginal infection of Candida albicans
Source: Data Brief. 2018 Mar 9;18:432–40. doi: 10.1016/j.dib.2018.03.006 (PMC5996161; doi:10.1016/j.dib.2018.03.006)
Supplement: Supplementary file 2 — Supplementary material Figure S1: Transcript level analysis of autophagy marker genes in wild-type C57BL/6 mice infected withC. albicans. Reverse Transcriptase PCR (RT-PCR) of LC3 (79 bp) (a), ATG5 (148 bp) (b) and LAMP1 (521 bp) (c) transcripts in vaginal cells isolated from wild-type C57BL/6 mice infected vaginally with C. albicans for different time points and their respective uninfected mice. 18s rRNA (150 bp) was used as the reference gene. Lane 1: 50 bp ladder, Lane 2: 7 days post-infection, Lane 3: 14 days post infection, Lane 4: 21 days post infection, Lane 5: 28 days post-infection, Lane 6: 35 days post-infection, Lane 7: 42 days post-infection, Lane 8: 49 days post-infection, Lane 9: 56 days post-infection, Lane 10: No template control. [file mmc2.docx]

**Supplementary Figure**

**1 2 3 4 5 6 7 8 9 10**


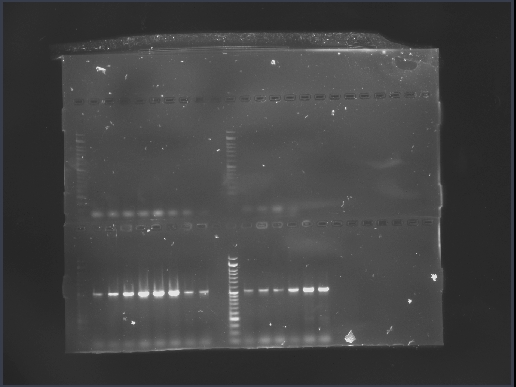

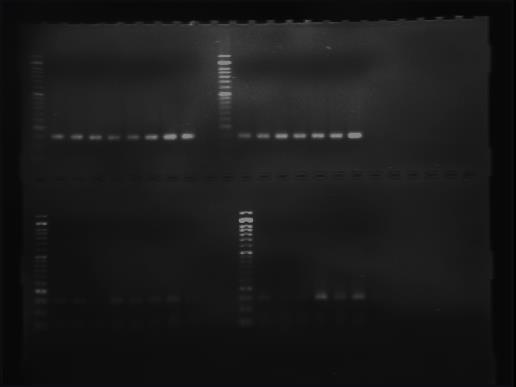


**Infected**

**18s**

**LC3**


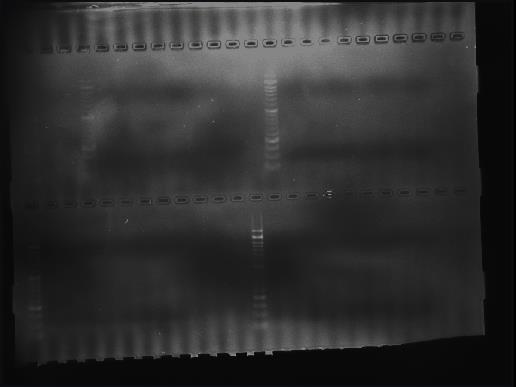

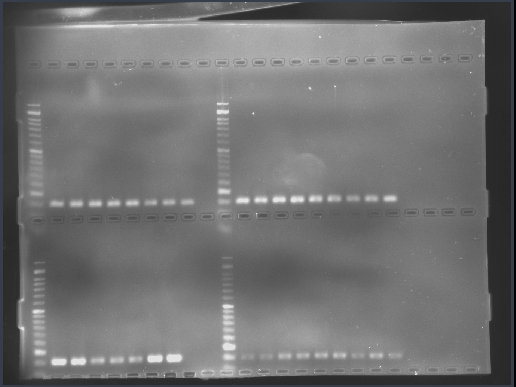


**Uninfected**

**18s**

**LC3**

**(a)**


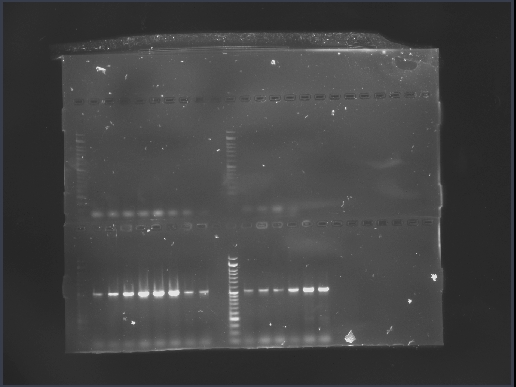

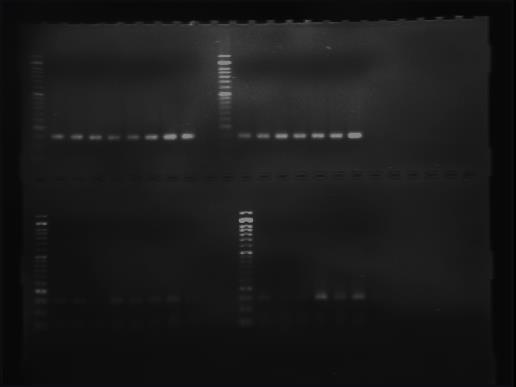


**18s**

**LAMP1**

**Infected**


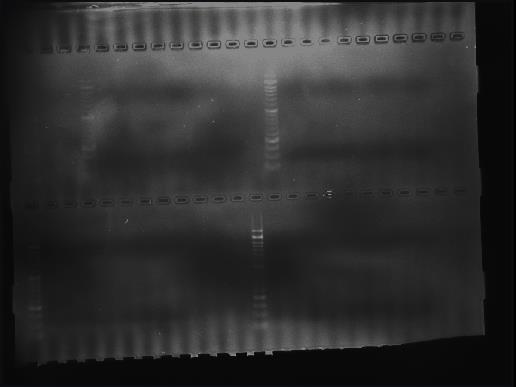

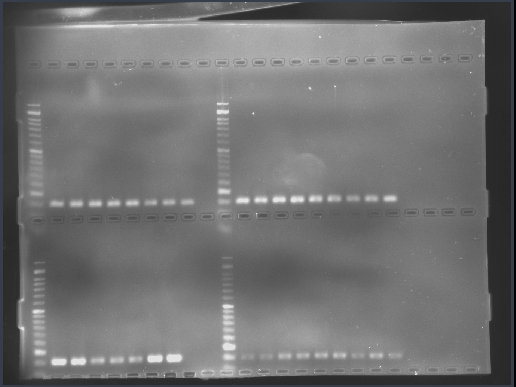


**18s**

**LAMP1**

**Uninfected**

**(c)**


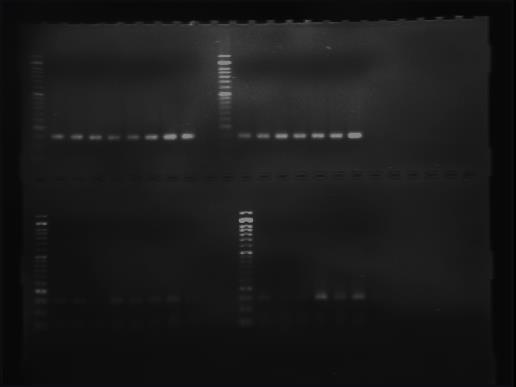


**18s**


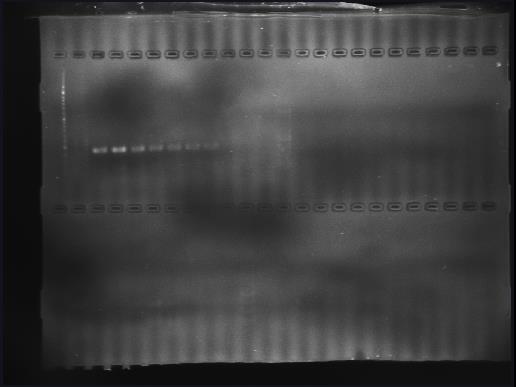


**ATG5**

**Infected**


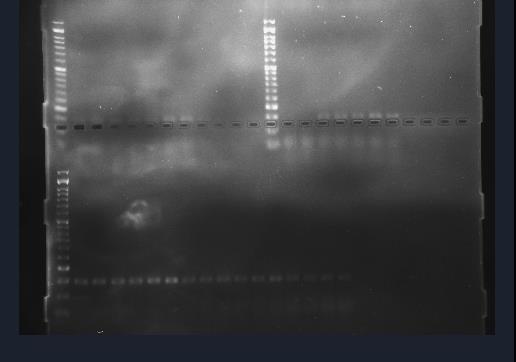

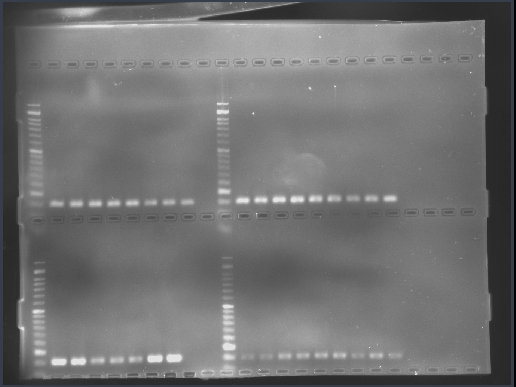


**Uninfected**

**18s**

**ATG5**

**(b)**

**Figure S1: Transcript level analysis of autophagy marker genes in wild-type C57BL/6 mice infected with *C. albicans*.** Reverse Transcriptase PCR (RT-PCR) of LC3 (79bp) (a), ATG5 (148bp) (b) and LAMP1 (521bp) (c) transcripts in vaginal cells isolated from wild-type C57BL/6 mice infected vaginally with *C. albicans* for different time points and their respective uninfected mice. 18s rRNA (150bp) was used as the reference gene. Lane 1: 50bp ladder, Lane 2: 7 days post infection, Lane 3: 14 days post infection, Lane 4: 21 days post infection, Lane 5: 28 days post infection, Lane 6: 35 days post infection, Lane 7: 42 days post infection, Lane 8: 49 days post infection, Lane 9: 56 days post infection, Lane 10: No template control.
